# Mr. Wallace on Sulphureous Fumigations in Rheumatism, and Diseases of the Skin

**Published:** 1821-12-01

**Authors:** 


*Observations on Sulphureous Fumigations, as a powerful Remedy in Rheumatism and Diseases of the Skin.* By William Wallace, M. R. I. A. Member of the Royal College of Surgeons in Ireland; one of the Surgeons to the Charitable Infirmary, Jervis Street; Surgeon to the Dublin Infirmary for curing Diseases of the Skin; Lecturer on Anatomy, &c. One vol. 8vo, pp. 92. Dublin, 1820.

Mr. Wallace appears to deplore the retardation of the arts and sciences, even in this enlightened æra, by the indifference or unwillingness with which new discoveries are frequently received, "more particularly when they are much in opposition to accustomed modes of thinking and acting, and when trouble and exertion are required for their application;" while at the same time, for want of sufficient zeal to examine into the foundation of opinions and practice, when first promulgated, "the errors of enthusiasts or the assertions of dissemblers are sometimes permitted to be propagated, and to have a long and powerful influence in retarding our enquiries after truth."

Our author has, for some years, directed his attention to diseases of the skin, in the Irish metropolis, where cutaneous defœdations are particularly prevalent. In 1818 he was enabled to found a dispensary for this class of complaints, and finding sulphureous fumigations of such great utility, he has, in this publication, attempted a succinct history of the origin of the invention—of its progress on the Continent—and of the investigations to which it had been submitted there, contenting himself *for the present* with adding, in conclusion, some general observations and reflections derived from his own experience in hospital and private practice. The greater part of the pamphlet before us consists of a history of the introduction and establishment of sulphureous vapour-baths in France, under the superintendance of Dr. Galés—and of translations of cases and observations by Dr. De Carro, of Vienna, published in 1819. We cannot go into an analysis of these cases, but we recommend a careful perusal of them to all those who have the means of employing the fumigations in question. We introduce the following description of the effects of this bath on the human frame, as it corresponds exactly with what we have witnessed ourselves at an establishment in this metropolis.

"The temperature of the apparatus should be such as, without creating uncomfortable feelings, will cause a copious flow of perspiration, about ten or fifteen minutes after the patient has entered the apparatus; and, as very different degrees of heat will be required for this purpose, dependent on the various circumstances of sex, age, temperament, or disease, no very determined rule can be laid down. For the most part, I have found a range from 100° to 120° of Ft. necessary. Sometimes, however, a lower temperature has answered the purpose; and, not unfrequently, patients have expressed themselves better pleased with one much higher.

"In general, when the patient has been in the apparatus about ten or fifteen minutes, we remark his face to become red, his eyes brilliant and more prominent, his pupils much dilated, the capillaries of the conjunctiva injected with red blood, and all the appearance of a high degree of excitement. This is soon followed by an effusion of perspiration; first in minute points, afterwards in larger drops, and, in some cases, it becomes so profuse as to trickle in a large quantity down the cheeks.

"If the surface of the patient, which is inclosed in the apparatus be now examined through one of the openings, it feels remarkably warm, and bathed in perspiration; which communicates to the hand a peculiar sensation, not of an oily nature, but astringent, almost as if we immersed our hands in a solution of sulphate of alumen. No doubt this is owing to the deposition of the sulphureous acid on the skin, and its combination with the secretion from the surface. At the same time, the pulse and respiration are a little accelerated; subject, however, to considerable variety, and the former feels full and strong.

"For the most part, when the patient has remained in the apparatus about thirty minutes, (the medium time for adults,) his situation becomes irksome, and he desires to come out. To these sensations we shall particularly attend, as they will afford the best principle to regulate the period of fumigation.

"In some very rare instances, a feeling of debility, of vertigo, and of sickness comes on, while the patient is in the apparatus. This is, however, as I have mentioned, extremely rare; and seldom advances to such an extent as to cause an interruption to the operation. It should, nevertheless, be attended to. If likely to arrive at a serious height, the fumigation must be terminated; and, when repeated, the patient must be kept a shorter time in the apparatus.

"When the operation is terminated, and that we have an opportunity of inspecting the surface of the patient, the vascularity of the skin is found to be extremely encreased; for not only are all the cutaneous capillaries, but also the superficial veins, particularly those of the extremities, distended with blood. The degree of this determination to the surface is nevertheless so subject to variety, dependent partly on the constitution, age, and disease of the patient, and partly on the temperature of the apparatus, and the quantity of sulphureous acid disengaged, that, while, on some occasions, the skin is as red as scarlet, on others, we do not observe more than a general faint blush.

"The quantity of fluid discharged from the skin, for the most part, creates greater astonishment than the redness of the surface; for the skin is not merely covered with general moisture, but oftentimes the seat and the bottom of the apparatus are perfectly wet with the fluid, that has dropped from the patient during the operation.

"This discharge from the surface, and the general and excessive excitement, are sometimes followed by a momentary feeling of exhaustion; but, on other occasions, the excitement produced appears to continue for hours, accompanied by a high complexion and vivacity of countenance, and gradually to go off without any feelings of debility or languor succeeding. Even in those cases, where this feeling of exhaustion is most remarkable, it generally ceases before the patient is dressed." 80.

The continental physicians (and we may add some of those who superintend sulphur fumigations in London) cause their patients to recline in a bed or on a couch for an hour or two after each operation. In the majority of cases, Mr. Wallace thinks, this is not necessary—on the contrary, he is disposed to believe that an opposite practice, viz. that of immediately dressing and taking some gentle exercise (weather permitting) on horse or foot, would be more beneficial. It occasionally happens that, before patients become habituated to the sulphur vapour bath, they complain of slight vertigo, watchfulness, and diminution of appetite. But these symptoms are fugitive, excepting in a very few cases which require a suspension of the operation.

"Frequently, however, a very opposite effect, even from the first fumigation, is observed, the appetite of the patient is improved, his rest becomes more tranquil and more profound, his alvine discharges more copious and regular, and a diminution takes place of any nervous excitement, that may have existed.

"Perhaps the most remarkable effect, observed to arise from the operation of sulphureous fumigation, is that of its causing, in some cases, a general desquamation of the cuticle, and in others, a peculiar papular eruption." 82.

This desquamation sometimes does not take place till several weeks after the bath is left off—proving, Mr. Wallace thinks, that these fumigations continue to influence the body, and modify its organic actions long after they are disused—and explaining, of course, why their beneficial influence on diseases is sometimes not apparent till after the remedy is left off. The symptom of desquamation is a very favourable one—generally indicative of the disease giving way to the remedy. The eruption (papular) sometimes produced by this process, bears some analogy to what is called the bath eruption, and is accompanied by some febrile excitement, smarting, and itching—always terminating in desquamation. Occasionally this occurrence interrupts, for a very few days, the treatment by fumigation. The following passage evinces candour on the part of our author.

> "In the course of our employment of this remedy, we shall occasionally be much disappointed. Cases, in which it is clearly indicated, and in which, from experience in others closely resembling them, we might have every reason to presume on its efficacy, will resist its operation. Occasionally, when we conceive, that a cure is almost accomplished, our hopes are dashed and disappointed, and an aggravation, at least of a temporary nature, of all the symptoms, occur. Sometimes, also, its beneficial effects, although certain and progressive, are remarkably slow. 84,

This, however, as Mr. Wallace justly observes, is no more than we might expect, in so obstinate a class of diseases as those of the skin.

In general Mr. Wallace regulates his conduct on the following principles, to wit:—if, after a few applications, the disease is rendered worse, instead of being benefitted, it will, for the most part, be useless to persevere. But if the application can be continued without any aggravation of the disease, he should persevere for a considerable time: for, occasionally when the fumigation is unequal to the removal of a disease, it renders it susceptible of being cured by medicines.

A contemplation of the phenomena produced by sulphureous fumigation, as described in the extract introduced, and as we have frequently witnessed, must convince us that the measure is a very powerful one—and one that, under judicious management, may be usefully employed in many other than cutaneous diseases. We hope therefore that these apparatuses will soon be erected in our public hospitals, and the extent of their utility thus ascertained.

A plan of the fumigating apparatus, and directions for using it, are appended to Mr. Wallace's work.

